# 3Cnet: pathogenicity prediction of human variants using multitask learning with evolutionary constraints

**DOI:** 10.1093/bioinformatics/btab529

**Published:** 2021-07-16

**Authors:** Dhong-Gun Won, Dong-Wook Kim, Junwoo Woo, Kyoungyeul Lee

**Affiliations:** Research and Development Center, 3billion, Seoul 06193, Republic of Korea; Research and Development Center, 3billion, Seoul 06193, Republic of Korea; Research and Development Center, 3billion, Seoul 06193, Republic of Korea; Research and Development Center, 3billion, Seoul 06193, Republic of Korea

## Abstract

**Motivation:**

Improvements in next-generation sequencing have enabled genome-based diagnosis for patients with genetic diseases. However, accurate interpretation of human variants requires knowledge from a number of clinical cases. In addition, manual analysis of each variant detected in a patient's genome requires enormous time and effort. To reduce the cost of diagnosis, various computational tools have been developed to predict the pathogenicity of human variants, but the shortage and bias of available clinical data can lead to overfitting of algorithms.

**Results:**

We developed a pathogenicity predictor, 3Cnet, that uses recurrent neural networks to analyze the amino acid context of human variants. As 3Cnet is trained on simulated variants reflecting evolutionary conservation and clinical data, it can find disease-causing variants in patient genomes with 2.2 times greater sensitivity than currently available tools, more effectively discovering pathogenic variants and thereby improving diagnosis rates.

**Availability and implementation:**

Codes (https://github.com/KyoungYeulLee/3Cnet/) and data (https://zenodo.org/record/4716879#.YIO-xqkzZH1) are freely available to non-commercial users.

**Supplementary information:**

Supplementary data are available at *Bioinformatics* online.

## 1 Introduction

Missense variants are those with which one amino acid in a protein is replaced by another amino acid due to a nucleotide change in a gene. Missense variants are common, corresponding to 83% of non-synonymous variants in the population, and in most cases, their pathogenicity is less severe than that of non-sense variants or frameshift variants (Lek *et al.*, 2016). Nevertheless, many genetic diseases, such as developmental disorders, heart malformations and many kinds of syndromic disorders, are caused by missense variants (Homsy *et al.*, 2015; Iossifov *et al.*, 2014; Jin *et al.*, 2017). Recent improvements in next-generation sequencing (NGS) have enabled the identification of massive numbers of variants in patients with genetic disorders. Given the frequent occurrence of missense variants, it is not surprising that they are commonly found in the genome of patients (Pérez-Palma *et al.*, 2019). However, the clinical pathogenicity of a missense variant is difficult to predict because it is necessary to comprehensively consider the effect of the variant on the protein, the cell, and, ultimately, the body (Gatz *et al.*, 2019). In addition, identifying a true disease-causing variant among many detected missense variants is crucial for diagnosis. Therefore, analyzing the effect of missense variants is an important and challenging problem in the clinic.

There are standard guidelines for diagnosing patients based on the interpretation of sequence variants as recommended by the American College of Medical Genetics and Genomics, that is, the ACMG guidelines (Richards *et al.*, 2015). Based on these guidelines, the pathogenicity of each variant in patients has been reported to the public database, ClinVar (Landrum *et al.*, 2016), as one of the following five classes: pathogenic (P), likely pathogenic (LP), uncertain significance (VUS), likely benign (LB) and benign (B). However, ClinVar includes fewer than 100 000 missense variants with known pathogenicity and reliable confidence. According to dbNSFP, the possible number of missense variants within the human genome is 82 755 468 (Liu *et al.*, 2011), indicating that the pathogenicity of missense variants is rarely known. Furthermore, considerable time and effort are needed to confirm a disease-causing variant and diagnose a patient, leading to a high failure rate of diagnosis and delays in treatment for patients (Amendola *et al.*, 2016). Due to the importance of missense variants in genetic diseases, there is an unmet need to determine the pathogenicity of VUS variants found in the patient genome (Ghosh *et al.*, 2017). If a prediction algorithm could predict the disease-causing variants in advance, it would considerably reduce the time and cost required for diagnosis. PP3 is one of the standards in the ACMG guidelines that applies to *in silico* assessments. The importance of PP3 is continuously growing because the assessment of missense variants depends largely on *in silico* prediction (Ghosh *et al.*, 2017).

In the field of computational genomics, various attempts have been made to develop artificial intelligence (AI)-based diagnostics using the rapidly increasing volume of genomic data. Some attempts have been made to predict the pathogenicity of variants using machine learning algorithms. As an example, REVEL uses a random forest algorithm that incorporates various pathogenicity predictors to build an integrated predictor for missense variants (Ioannidis *et al.*, 2016). CADD is another ensemble method that uses linear regression to integrate different scoring tools (Rentzsch *et al.*, 2019). FATHMM applies multiple sequence alignments (MSAs) to recognize evolutionary constraints (Shihab *et al.*, 2013). VEST4 (Carter *et al.*, 2013), POLYPHEN2 (Adzhubei *et al.*, 2010) and SIFT (Kumar *et al.*, 2009) are other well-known prediction tools used to predict changes in protein functionality based on random forest, naive Bayes and statistical methods, respectively. DANN was the first artificial neural network used to predict the pathogenicity of non-synonymous variants based on 949 features (Quang *et al.*, 2015). However, there have been issues with circularity as some conventional predictors use scores from other tools (Grimm *et al.*, 2015). Circularity can lead to the overlap of training data between many tools, consequently resulting in overfitting. In addition, the shortage of clinical data causes overfitting of AI machines to previous knowledge. However, there have been novel attempts to solve this problem by applying sequence-based pathogenicity prediction. PrimateAI compares the sequences of wild-type and mutant proteins to identify differences and estimate the probable pathogenicity of variants using a convolutional neural network (CNN) (Sundaram *et al.*, 2018). Approaches that utilize protein sequences for pathogenicity prediction are promising because they can consider the context of amino acid sequences and avoid overfitting to previous knowledge.

We developed an algorithm that uses recurrent neural networks (RNNs) to predict pathogenicity based on the protein sequence around the variant. To avoid overfitting for available clinical data, we utilized variants from other sources of data, such as common variants frequently observed in the general human population (Song *et al.*, 2016), and conservation data, which refers to the simulated variants that we generated based on evolutionary conservation information. In this study, we utilized multi-task learning (Ruder, 2017) to integrate three different types of data as follows: clinical data, common variants and conservation data. We found that training on simulated variants improved the prediction accuracy of pathogenicity predictors by reducing overfitting to known variants. We also utilized features representing the physical and biochemical changes in proteins upon amino acid mutation from the public database, SNVBox, to assemble a comprehensive algorithm (Wong *et al.*, 2011). The resulting pathogenicity predictor 3Cnet evaluated the impact of missense variants more accurately than any other pathogenicity predictor (PR-AUC = 0.916) and identified the disease-causing variants of genetic disease patients with 2.2 times greater sensitivity than other predictors (top-1 recall = 14.5%). 3Cnet is the first RNN-based pathogenicity predictor to learn the effect of variants in the context of protein sequences. 3Cnet can also predict the pathogenicity of other non-synonymous variants including start lost, stop gain, deletion and frameshift variants with better accuracy compared to others (PR-AUC = 0.986).

## 2 System and methods

### 2.1 Generation of clinical data from the ClinVar database

We first curated 72 470 missense variants from ClinVar (released in April 2020) with known molecular consequences and reliable review status. For this purpose, we collected variants in which the molecular consequence was ‘missense variant’ and excluded those with unreliable review status, such as ‘no assertion for the individual variant’, ‘no assertion criteria provided’ and ‘no assertion provided’. As our prediction algorithm utilizes the protein sequence around the variant site as an input feature; each variant is represented by the Human Genome Variation Society (HGVS) term, in which the transcript ID and the variant information are given (den Dunnen *et al.*, 2016). The transcript ID was the canonical transcript in the RefSeq database (version GRCh37) (Pruitt *et al.*, 2005). Then, each missense variant was transformed to data representing a protein sequence composed of 201 amino acids centred around the variant site (Sundaram *et al.*, 2018). Sequence data for both wild-type and mutant proteins were generated to compare the difference in the context of amino acid sequences.

In some cases, there were multiple reports for a single variant, with possible conflict (e.g. one was pathogenic, whereas another was benign). Therefore, the pathogenicity of each variant was determined by integrating pathogenicity reports from ClinVar for the same variant. We used the following five labels for pathogenicity: pathogenic (P), likely pathogenic (LP), variants with uncertain significance (VUS), likely benign (LB) and benign (B). We set a standard to define pathogenicity for each variant from multiple reports. When there were any reports indicating that a variant was pathogenic or likely pathogenic, we considered the variant to be pathogenic, except for the cases in which there were contrary reports. Similarly, a variant with any reports indicating that the variant was benign or likely benign, we considered the variant to be benign. We removed variants with contrary reports and variants with reports of VUS only. As a result, we obtained 22 337 pathogenic variants and 50 133 benign variants from the ClinVar database. The input features of the clinical data are sequence representations of wild-type protein and mutant protein, and the output feature is a binary label indicating pathogenicity.

For the variants other than missense variants, including start lost, stop gain, deletion and frameshift variants, the center of the sequence was set to the residue where the truncation started or ended. For example, in the case of a start lost variant ‘p.Gly2_Met46del’, the residue 46 which is the new initiation site has become the center of the sequence data. The truncated region of the mutated sequences was filled with zeros (zero padding). In the case of a stop gain variant, the new termination site was used as the center and the region beyond the site was filled with zeros for the mutated sequence. Frameshift variants were treated the same with stop gain variants and the sites where insertion/deletion occurred were considered the termination sites. For other deletion variants, the site where the deletion started was used as the center. Overall, 147 034 pathogenic variants and 3995 benign variants were found and transformed into sequence data. Note that these variants were more likely to be pathogenic compared to missense variants.

### 2.2 Augmentation of clinical data with common variants in gnomAD

We mined the gnomAD database to obtain missense variants frequently observed in the general population, namely, common variants (Karczewski *et al.*, 2017). Such variants are found in the genomes of a number of ordinary people, suggesting that they are benign variants. Common variants were used as benign variants to train the predictors and obtain better precision by reducing false positives (Gilissen *et al.*, 2012). First, common variants with allele frequencies (AFs) higher than 0.1% were collected. Then, those variants were represented by the HGVS term based on the canonical transcripts as had been done for the ClinVar data. Only variants not included in the ClinVar data were curated to avoid conflicts and overfitting due to duplicate samples. As a result, 60 614 common variants were found and labelled benign. As the number of benign variants, including data from ClinVar, was much larger than the number of pathogenic variants (22 337 versus 110 747), pathogenic samples were augmented 4-fold during training for balance. After removing a few transcripts inconsistent with the reference sequence of HG19 (Church *et al.*, 2011), 18 942 unique transcripts were included in the curated data in total.

### 2.3 Generation of conservation data using MSA

For the 53 998 transcripts included in the RefSeq database, MSAs were constructed to show the evolutionary conservation patterns of those proteins. For each transcript, the sequence was transformed into FASTA format. Then, a hidden Markov model-based algorithm, HHblits (Remmert *et al.*, 2011), built MSAs from the query sequence using UniRef30 (version 2020.02) as the sequence database (Suzek *et al.*, 2007). These MSA results were utilized for two purposes as follows: to derive input features describing evolutionary conservation and to generate simulated variants reflecting evolutionary constraints. Among the sequences aligned with the query sequence, only sequences with over 30% identity and 80% overlap with the query were retained. In addition, only residues aligned with more than 10 residues from other sequences were regarded as reliable alignments and utilized for the following processes.

Only 133 084 variants were collected from clinical data, even when common variants were included as benign samples. Because this number was not sufficient for training a deep neural network with more than 10 000 features as sequence inputs, more variants were needed to train our model in an unbiased way to avoid overfitting, which can occur when a dataset is too small. Therefore, we generated simulated variants based on the amino acid frequency at each residue in the alignment. First, we randomly created variants at each residue of the transcripts, considering the trinucleotide context in their genomes (Sundaram *et al.*, 2018). We defined positions where one amino acid was represented in over 50% of aligned sequences as ‘conserved.’ Among those variants, we defined the variants found at conserved residues as pathogenic-like variants. Frequently found variants with a ratio higher than 10% out of aligned amino acids were defined as benign-like variants. We then randomly selected 10% of all possible simulated variants to maintain a reasonable training cost. We referred to those variants as conservation data and utilized them for multi-task learning along with clinical data to train the models. The resulting numbers of variants for each type of data are summarized in Supplementary Table S1.

## 3 Algorithm

### 3.1 Network architecture of the pathogenicity predictor

**Fig. 1.**
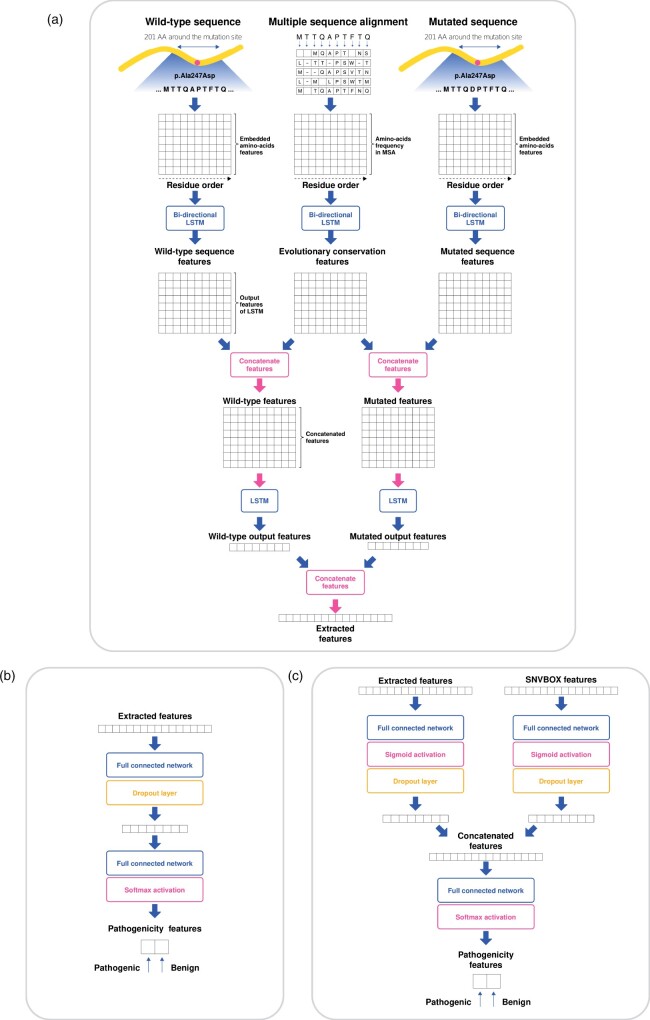
The architecture of the recurrent neural network to train variant pathogenicity based on protein sequences. (**a**) The feature extractor contains two parallel layers of long short-term memory (LSTM) networks. The features of the wild-type sequence, mutated sequence and MSA are merged and processed to become a vector, known as the extracted features. (**b**) The pathogenicity classifier is composed of two fully connected layers that determine the binary pathogenicity of a variant from the extracted features. (**c**) The pathogenicity classifier utilizes SNVBox features and the extracted features by combining these two features through sigmoid activation followed by concatenation

The pathogenicity predictor we built is composed of the following two sequential modules: a feature extractor and a pathogenicity classifier (Fig. 1). The feature extractor is composed of two parallel layers utilizing long short-term memory (LSTM) networks, a type of RNN (Hochreiter and Schmidhuber, 1997). The first layer consists of bidirectional LSTM networks that independently feature three different feature matrices as follows: wild-type sequence feature matrix, MSA feature matrix and mutant sequence feature matrix (Supplementary Note S1). The output feature matrices of LSTM consider the context of the sequences through the recurrent networks to determine the influence of one amino acid on the other amino acids. Then, the output matrix from the wild-type sequence and that of MSA are merged to produce a concatenated feature matrix. Features for the same residue are concatenated such that the network can compare the amino acid of the sequence with evolutionarily conserved amino acids at that residue. The output matrices of the mutant sequence and the MSA are also merged. Then, the two concatenated features (wild-type and mutant) are featured once more using LSTM networks. At this point, however, only the last feature vector from the recurrent network remains. Finally, the output feature vectors of the wild-type and the mutant are concatenated to become an extracted feature vector.

The subsequent pathogenicity classifier is composed of two fully connected (FC) layers. The first FC layer is expected to extract the difference between the wild-type and mutant sequences as a feature vector. A dropout layer is applied to the first layer to avoid overfitting. Then, the final FC layer is used to decide the pathogenicity of a variant by applying softmax activation to classify the variant as pathogenic or benign. The binary cross-entropy between labels and the predicted classes become the loss function of the network. In the case of models using SNVBox features as additional inputs, the feature vector is merged with the extracted feature vector. Before concatenation, each feature vector passes through a separate FC layer and sigmoid activation to address the scale difference between the two different input features. Then, the merged features are used to determine the pathogenicity of the variant by the following FC layer.

### 3.2 Optimizing the pathogenicity predictor using various training data

**Fig. 2.**
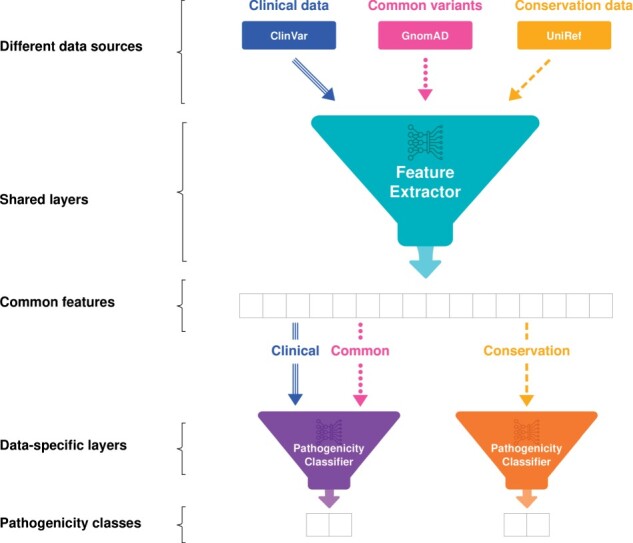
Multi-task learning between clinical data and conservation data. The feature extractor is trained by all data sources, including clinical data from the ClinVar database, common variants from the gnomAD database, and conservation data generated based on the UniRef database. Therefore, the extracted features become common features for different types of data. In contrast, pathogenicity classifiers are separated for specific data types. The clinical data and the common variants are used to train the pathogenicity classifier, while the other pathogenicity classifier is trained by conservation data. After training, the pathogenicity of a variant is determined by the classifier trained by clinical data

First, we trained a prediction model using only clinical data curated from the ClinVar database. In addition, we trained a model with ClinVar variants along with common variants obtained from the gnomAD database, which were used as benign samples. In another model, conservation data generated based on MSA were used to train the predictor. As patterns of evolutionary conservation are widely used to predict the pathogenicity of genomic variants, a model trained with conservation data only might be able to predict pathogenicity to some extent (Adzhubei *et al.*, 2010; Rentzsch *et al.*, 2019; Shihab *et al.*, 2013). We also aimed to transfer the knowledge obtained from evolutionary conservation to train a model with clinical data without overfitting. With multi-task learning (Ruder, 2017), the model utilizes a shared feature extraction network for both clinical data and conservation data, but an independent pathogenicity classifier is used for each (Fig. 2). The prediction scores for the multi-task model were from the pathogenicity classifier trained on clinical data rather than the classifier trained on conservation data.

To select the optimized model among those various models, we verified the performance of the models using 5-fold cross-validation of the clinical data. Only clinical data from ClinVar, which were confirmed by ACMG guidelines, were used as the test set for cross-validation. Note that none of the common variants overlapped with the ClinVar database because duplicates were removed. Receiver operating characteristic (ROC) curves and precision-recall (PR) curves were measured, and the area under the curve (AUC) was calculated. Based on ROC-AUC and PR-AUC, we selected the optimal prediction model for discovering the pathogenicity of variants. In this study, the hyperparameters of the network were not optimized for the data to allow comparison of the different models without bias.

### 3.3 Combining SNVBox features for pathogenicity prediction

SNVBox is a database providing features that predict the biological impact of single nucleotide variations (SNVs) (Wong *et al.*, 2011). SNVBox comprises 85 structure-based, position-specific and exon-specific features that offer general information about proteins and amino acid substitutions of SNVs. For example, SNVBox includes physicochemical properties of amino acid substitution, such as change, volume, hydrophobicity and polarity. SNVBox also includes specific features of the protein, such as active sites, known motifs and protein-protein interactions. Training SNVBox features along with sequence data may improve the lack of information about the significance of proteins and regions where the variants occur. Some genes, such as paralogues, may have similar sequences but different functions or pathogenicity of variants. Utilizing protein-specific features may enable distinguishing biological differences between similar genes. Therefore, we expected a synergetic effect between the extracted features derived from sequence inputs and the SNVBox features, implying physical and chemical states of the proteins. In addition, as SNVBox features do not depend on other scoring methods, these features are free from the circularity problem. The SNVBox features were integrated with the extracted features and then used to predict pathogenicity (Fig. 1c). For variants that could not be found in the SNVBox database, the feature vectors were filled with zeros. The model network utilizing SNVBox features was trained by multi-task learning and named 3Cnet.

## 4 Results

### 4.1 Improving pathogenicity predictor using augmented variants

**Fig. 3.**
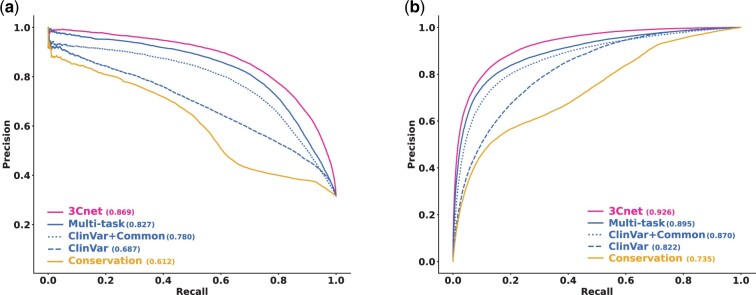
Cross-validation of internal ClinVar variants for different models using the recurrent neural network. (**a**) PR curve for cross-validation. Conservation (solid yellow) indicates the model trained by conservation data. ClinVar (dashed blue) indicates the model trained using only clinical data from the ClinVar database. ClinVar+Common (dotted blue) indicates the model trained by ClinVar data along with the common variant. The multi-task (solid blue) model is trained by multi-task learning between clinical data and conservation data. 3Cnet (solid magenta) is the model trained by multi-task learning with pathogenicity classifiers that utilize SNVBox features. (**b**) ROC curve for cross-validation

Figure 3 compares the performance of various pathogenicity predictors in terms of ROC-AUC and PR-AUC. While the model trained with ClinVar variants only had a PR-AUC of 0.687, the model trained by both ClinVar variants and common variants had a PR-AUC of 0.780. The results showed that the inclusion of common variants as benign variants contributed to improving the accuracy of the prediction model. In addition, even though the predictor trained on conservation data only had relatively low performance (PR-AUC = 0.612), the predictor trained by multi-task learning with both clinical data and conservation data (PR-AUC = 0.827) showed better pathogenicity prediction performance compared to the model trained on clinical data only. Overall, the predictor integrated with SNVBox features, 3Cnet, demonstrated the best pathogenicity prediction performance (PR-AUC = 0.869).

### 4.2 External validation for independent ClinVar variants

To compare other prediction tools with 3Cnet, we built an external dataset independent of the training dataset used for cross-validation (Bleeker *et al.*, 2003). As the ClinVar dataset that we used as training data was released in April 2020, we utilized novel variants in ClinVar released in August 2020 as external data. All the variants reported in the previous ClinVar dataset were excluded from the external validation set. The reported pathogenicity and prediction results of those overlapping variants are summarized in Supplementary Table S2. In addition, only variants with scores annotated for all tools consisting of REVEL, VEST4, SIFT, PolyPhen, PrimateAI, CADD, FATHMM, DANN and 3Cnet remained. Pathogenicity scores for each tool were collected from dbNSFP (version 4.0), except for PrimateAI, for which scores were from the Illumina database, Basespace. The external validation set needed to be independent not only of the training data we used for 3Cnet but also of the training data used for other tools for fair comparison. Even though we were unable to specify every single variant used to train other tools due to the use of the commercially available database, HGMD (Stenson *et al.*, 2003) and the circularity problem, we reasonably infer that the external variants were mostly exclusive from the variants used to train other tools as dbNSFP v4.0 (May 2020) was formerly released.

**Fig. 4.**
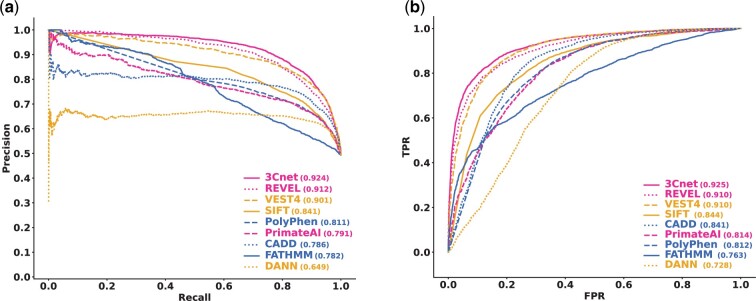
Validation performance of external ClinVar variants and comparison with other pathogenicity prediction tools. (**a**) PR curve for external validation. 3Cnet showed the best performance for the independent clinical data. REVEL, an ensemble model using scores from many prediction tools, was the second best followed by VEST4, which utilizes SNVBox features for prediction. The performance of PrimateAI was the best among previous deep learning-based algorithms. (**b**) ROC curve for external validation

Consequently, we collected 6298 pathogenic variants and 6468 benign variants as external validation data. We tested the performance of 3Cnet using the external validation set and compared it with those of other pathogenicity prediction algorithms. Figure 4 shows that 3Cnet (PR-AUC = 0.924) showed the best pathogenicity prediction performance among all the tested pathogenicity predictors, including REVEL (PR-AUC = 0.912), VEST4 (PR-AUC = 0.901), SIFT (PR-AUC = 0.841), Polyphen2 (PR-AUC = 0.811), PrimateAI (PR-AUC = 0.791), CADD (PR-AUC = 0.786), FATHMM (PR-AUC = 0.782) and DANN (PR-AUC = 0.649), which are currently widely used to confirm variant pathogenicity. These results indicated that 3Cnet classifies the pathogenicity of variants more accurately than previous methods without utilizing scores from other algorithms.

**Fig. 5.**
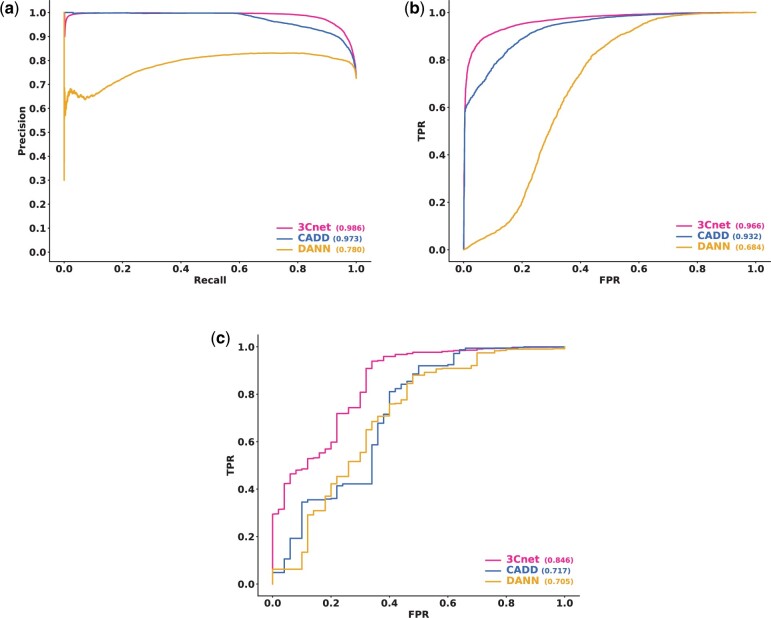
External validation performance for non-synonymous variants including start lost, stop gain, deletion and frameshift variants. (**a**) PR curve for non-synonymous variants. (**b**) ROC curve for non-synonymous variants. (**c**) ROC curve for non-synonymous variants except for missense variants

We also tried to see whether 3Cnet can train and assess the pathogenicity of non-synonymous variants other than missense variants. Human variants such as start lost, stop gain, deletion and frameshift variants have a high probability of being pathogenic through loss of function. We trained 3Cnet not only with the missense variants but also with those variants. Integrated non-synonymous variants consisted of 169 371 pathogenic variants and 54 128 benign variants. Also, we included common variants as benign variants with AF > 0.1% from gnomAD database. In total, 169 371 pathogenic variants and 114 743 benign variants were trained to build the test model. We collected external non-synonymous variants from ClinVar (August 2020) by excluding all the trained variants from the data. Unfortunately, only CADD and DANN was able to assess those variants among algorithms in dbNSFP (Fig. 5). For the external non-synonymous variants, consisting of 17 130 pathogenic variants and 6518 benign variants, we compared the performance of 3Cnet (PR-AUC = 0.986), CADD (PR-AUC = 0.973) and DANN (PR-AUC = 0.780). Also, we compared those algorithms using non-synonymous variants without missense variants. 3Cnet (ROC-AUC = 0.846) showed superior performance not only for missense variants but also for other non-synonymous variants compared to CADD (ROC-AUC = 0.717) and DANN (ROC-AUC = 0.705) (Fig. 5c). PR-AUC was consistently high for all the tools (0.999 for 3Cnet, 0.998 for CADD, 0.997 for DANN) because of the large number of pathogenic variants (10 832) compared to benign variants (50). Therefore, the PR curve was not shown in this case.

### 4.3 Assessment of missense variants of patients from confirmed cases

We also assessed the effectiveness of 3Cnet scores for distinguishing disease-causing variants from other missense variants in patients. Such an application is highly practical in clinical cases for the discovery of the disease-causing variant(s) among the large number of missense variants found in the genome of a patient. We obtained the missense variants found in the genomes of 111 patients with rare genetic disease who were diagnosed based on the ACMG guidelines (Seo *et al.*, 2020). The disease-causing variants were confirmed by medical doctors, and other missense variants from those patients were curated as non-causing variants. To test a reasonable number of variants, we removed variants with an AF > 0.1% in the general population. As some patients with autosomal recessive diseases had two disease-causing variants, 186 disease-causing variants and 54 496 non-causal variants were examined.

**Fig. 6.**
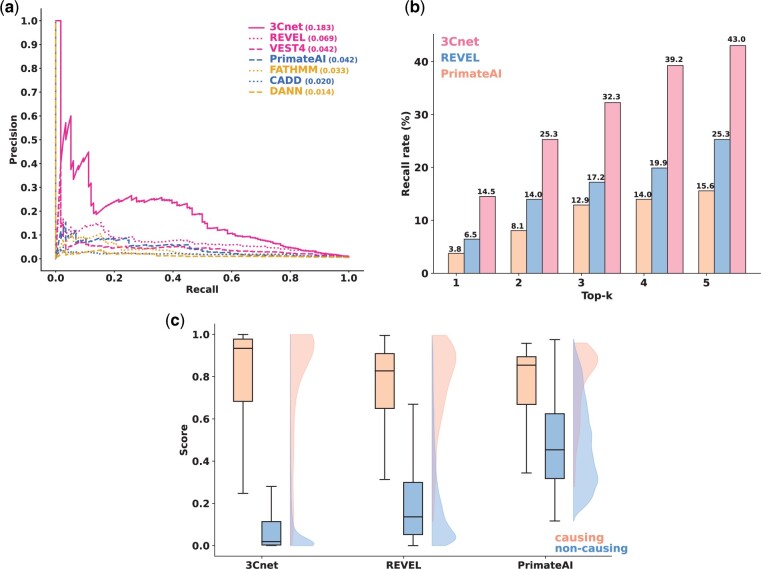
Discriminating disease-causing variants from other missense variants in the patient genome. (**a**) PR curve for classifying disease-causing variants and non-causal variants. (**b**) The top-k recall rate implies the probability of determining the true disease-causing variant(s) among the top ranked variants using prediction scores. This number is important because the diagnosis rate of patients is closely related to the recall rate. (**c**) Score distribution of different algorithms for disease-causing variants and non-causal variants. A smaller proportion of the uncertain area with similar scores for disease-causing variants and non-causing variants indicates a higher resolution of the scoring scheme for dividing these variants

We compared the PR curve of 3Cnet to those of REVEL, VEST4, PrimateAI, CADD, FATHMM and DANN (Fig. 6a). SIFT and Polyphen2 were excluded because they had too many variants with maximum pathogenicity scores (2210 variants for SIFT and 3451 variants for Polyphen2), which prevented a proper PR curve to be drawn (Supplementary Note S2). The results showed that 3Cnet identified disease-causing variants more effectively (PR-AUC = 0.183) than REVEL (PR-AUC = 0.069), VEST4 (PR-AUC = 0.042), PrimateAI (PR-AUC = 0.042), FATHMM (PR-AUC = 0.033), CADD (PR-AUC = 0.020) and DANN (PR-AUC = 0.014). ROC curves were not compared in this case due to the large imbalance between positives and negatives.

In addition, we compared the recall rate of the disease-causing variants among the top-k variants for each patient for k values ranging from 1 to 5, representing the probability of finding the true disease-causing variant from the genome of a patient (Fig. 6b). We selected REVEL and PrimateAI for comparison because REVEL was identified as the second-best tool and PrimateAI was the best model among previous tools using deep learning and it utilizes amino acid sequences similar to 3Cnet. The top-k recall of 3Cnet (43% for top-5) was considerably high, representing the ability to identify disease-causing variants in patients. Such results implied that 3Cnet would be useful for the practical purpose of diagnosing genetic disease in clinical cases. In addition, the score distributions for disease-causing variants and non-causal variants were compared using box plots (Fig. 6c). The distribution of 3Cnet scores showed less uncertainty compared to others, enabling causal variants to be more clearly distinguished from benign variants to reduce VUS burden (Ghosh *et al.*, 2017).

### 4.4 Finding novel pathogenic variants using 3Cnet scores

We also used the 3Cnet scores to discover novel pathogenic variants within the genome of patients. For some of the rare disease patients, known pathogenic variants were not found in the genomes, but their symptoms were similar to those of specific rare genetic diseases (Seo *et al.*, 2020). We speculated the existence of new missense variants not reported in the ClinVar database but with some level of pathogenicity. We scored the rare missense variants (AF < 0.1%) found in the genome of these patients and closely assessed the variants with 3Cnet scores greater than 0.9. We excluded variants with REVEL scores greater than 0.75 as they could be discovered without the help of 3Cnet. The threshold for the REVEL scores corresponds to the stringent threshold proposed by Ioannidis *et al.* (2016) As a result, we identified seven novel missense variants whose pathogenicity had never been reported to ClinVar but are likely to be pathogenic according to the ACMG guidelines (Table 1). Remarkably, there was a VUS variant (NP_056651.1: p.Gly54Glu) that could be classified as likely pathogenic only if the 3Cnet score was used as evidence for the PP3 rule. For practical use of 3Cnet for PP3 evidence, we recommend 0.75 for the moderate threshold of 3Cnet scores (Supplementary Note S3).

**Table 1.** Novel pathogenic variants discovered using 3Cnet and assessed based on the ACMG guidelines

HGVSp	Phenotype OMIM ID	Assigned ACMG rules	ACMG class	3Cnet score
NP_056651.1:p.Gly54Glu	617710	PM2, PM3, PP2	VUS^a^	0.990
NP_066287.2:p.Leu1660Val	613721	PS2, PM1, PM2	LP	0.979
NP_733842.2:p.Trp1725Ser	263200	PM2, PM3	VUS^b^	0.944
NP_001420.2:p.Arg1829Pro	613684	PS2, PM1, PM2	LP	0.907
NP_001005463.1:p.Asn197Asp	617330	PS2, PM2	LP	0.978
NP_006006.3:p.Leu1049Arg	614607	PS2, PM2	LP	0.995
NP_061947.1:p.Gly262Ser	616589	PS2, PM2, PP2	LP	0.993

a The ACMG class (VUS) could be changed to likely pathogenic (LP) only if the 3Cnet score was applied for PP3.

b The patient diagnosed with autosomal recessive disease (polycystic kidney disease 4) had trans variants, and the other variant was classified as LP.

## 5 Implementation and availability

The data that support the findings of this study are available at https://zenodo.org/record/4716879#.YIO-xqkzZH1. The data include missense variants used to train 3Cnet (variant_data), sequence data transformed from the variant data (sequence_data), SNVBox features for each variant (SNVBOX_features), and MSAs for protein sequences in the RefSeq database (msa_data.tar.gz). In addition, the prediction scores of different tools for external ClinVar data and patient data from confirmed cases are also provided (validation_result). The Python codes used for this study are available at https://github.com/KyoungYeulLee/3Cnet. They include the codes to build the sequence data from variant data to train neural networks (featurize) and the codes used to build and test the prediction network (model).

## 6 Discussion

Pathogenicity prediction using deep learning has had limitations for clinical use due to its low performance compared to conventional methods, which may be because deep neural networks requiring massive amounts of data for effective training. In general, available clinical data are insufficient for training deep neural networks without bias (Taylor and Nitschke, 2017). Indeed, when a deep neural network is trained solely on clinical data from ClinVar, the model is overfitted to the data and is unable to interpret novel pathogenic variants. Nevertheless, the usage of clinical data from patients diagnosed based on the ACMG guidelines is essential to build accurate pathogenicity predictors. We overcame this limitation of deep neural networks by taking advantage of the knowledge obtained from evolutionary conservation of protein sequences. Our conservation dataset consisted of 4 018 149 artificial variants generated based on MSA. Although the pathogenicity of the simulated variants was not confirmed by clinical analysis, such data could help to avoid overfitting to clinical data and contribute essential features reflecting the evolutionary constraints on the proteins.

The application of 3Cnet varies depending on the desired purpose. One application is finding the true disease-causing variant in the genome of a patient. On average, a given patient genome contains approximately 100–400 rare missense variants (Auton *et al.*, 2015). Among those variants, only one or two variants will directly cause the symptoms, while many of the others will be benign. Therefore, 3Cnet can be used to narrow down the candidate variants based on predicted pathogenicity, thereby reducing the time and cost spent for diagnosis. In addition, scoring variants using 3Cnet can be used to identify novel gene-disease associations. Some genes may not be linked to any diseases in OMIM, a gene-disease mapping database (Amberger and Hamosh, 2017), but they might have variants that are related to a disease. If multiple variants with high pathogenicity scores are found in a single gene and the symptoms of patients with those variants are similar to those of a specific disease, the gene can be hypothesized as correlating with the disease. Finally, the features extracted from the 3Cnet network can be utilized to train other deep learning models in which the pathological impacts of variants are important, for example, to predict the functional domains of genes based on their sequence.

3Cnet was trained by the set of benign variants mainly from public databases such as ClinVar and gnomAD, a composition that is not free from bias. 3Cnet could misunderstand some benign variants as pathogenic if such variants were not reported at the databases. Therefore, using 3Cnet to identify benign human variants needs to be careful and rigorous validation should be followed. Another shortage of 3Cnet is that it currently only takes 201 residues around the mutation site. Some of the proteins in human body have longer sequences, in which case, the length of input sequences may not be sufficient. Nevertheless, 3Cnet is the first RNN-based deep learning model to utilize sequence inputs to predict pathogenicity. We could use attention-based networks, such as transformers, rather than RNNs to train the model. As a popular technique for natural language processing, transformers might be able to train the context of protein sequences and associate distant amino acids using self-attention layers (Vaswani *et al.*, 2017).

## Supplementary Material

btab529_Supplementary_Data
