# Supplementary material for: 3Cnet: pathogenicity prediction of human variants using multitask learning with evolutionary constraints
Source: Bioinformatics. 2021 Jul 16;37(24):4626–34. doi: 10.1093/bioinformatics/btab529 (PMC8665754; doi:10.1093/bioinformatics/btab529)
Supplement: btab529_Supplementary_Data [file btab529_supplementary_data.zip › supplementary_information.docx]

**Supplementary table 1. The number of pathogenic and benign variants for each type of data**

| Data type | Data source | Pathogenic variants | Benign variants |
| --- | --- | --- | --- |
| Clinical data | ClinVar | 22,337 | 50,133 |
| Common variants | GnomAD | 0 | 60,614 |
| Conservation data ^1^ | UniRef30 | 2,507,367 | 1,510,782 |
| Non-synonymous data | ClinVar | 169,371 | 54,128 |
| External missense data | ClinVar | 6,298 | 6,468 |
| External non-synonymous data | ClinVar | 17,130 | 6,518 |
| Patient data ^2^ | Clinical cases | 186 | 54,496 |

Even though the variants were used as pathogenic variants and benign variants for training and/or testing, the pathogenicity of some variants is not strictly confirmed. 1) In the case of conservation data, the variants are generated based on MSA, therefore those variants were named pathogenic-like variants and benign-like variants. 2) The benign variants used for patient data are non-causal variants, that is, rare missense variants from the genome of patients except for the disease-causing variant(s). Therefore, those non-causal variants are not necessarily benign. Non-synonymous data indicates the variants including missense, start lost, stop gain, deletion, and frameshift variants.

**Supplementary Note 1. Featurization of sequences and multiple sequence alignments**

To train deep neural networks, each variant needed to be transformed into real feature values that represent the amino acid sequence around the variant site. A sequence can be interpreted as sequential inputs of amino acids, and each type of amino acid has a unique property that could be represented as a feature vector. We applied feature embedding to represent the property of an amino acid in the form of a feature vector. As a result, a sequence was transformed into a feature matrix filled with sequential feature vectors representing amino acids (Supplementary figure 1). As the length of the overall sequences varies depending on the transcripts, we considered only the 201 residues around the variant site (previous 100 AAs to following 100 AAs) to build the data. If the variant site was too close to the start point or endpoint of the sequence, such that some positions in the data were not represented by any amino acid, the empty positions were filled with vectors with zeros (zero padding). Both the wild-type sequence and mutated sequence were transformed into feature matrices and used as input features.

Along with the transcript sequence data, we made use of the conservation patterns in MSA as input features. As such features can represent evolutionary constraints imposed on the residues of transcripts, they could be crucial for pathogenicity prediction. Therefore, each residue in the sequence was imposed with a vector containing the frequency at which that amino acid was found at that residue in the MSA (Supplementary figure 2). For those residues for which the number of aligned sequences was less than 10, the values of the vector were filled with zeros. In total, 8,726,858 residues from the 18,942 transcripts were imposed with ratio vectors, and 3,693,654 residues were padded with zeros. As for the sequence data, we selected 201 residues around the variant site and built a feature matrix for those residues. The feature matrix was then used as an input feature to train the pathogenicity predictor.


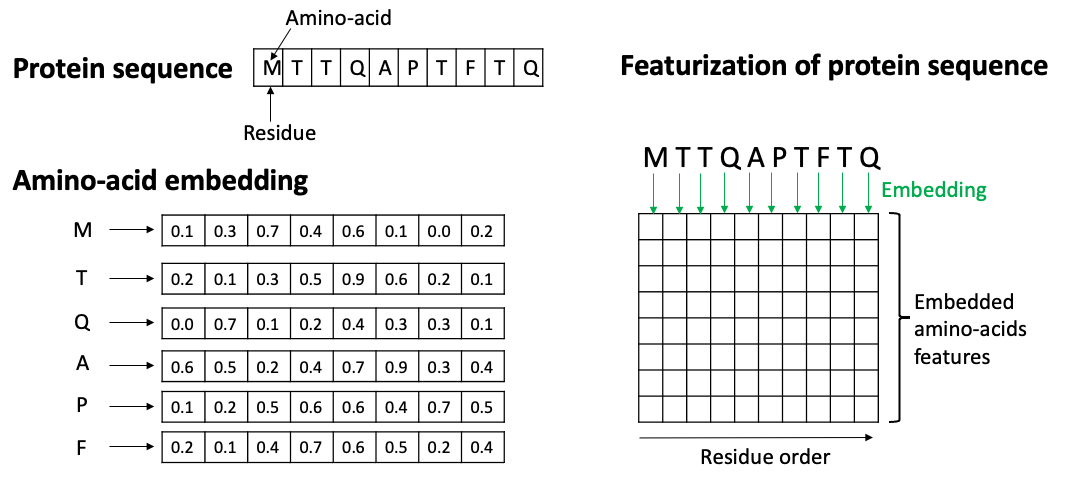


**Supplementary Figure 1. Transformation of a protein sequence into a feature matrix.** Each variant was transformed into the real value features which represent the amino-acid sequence around the mutation site. A sequence can be interpreted as sequential inputs of amino acids. Each type of amino-acid could have unique traits represented as a feature vector. We applied feature embedding to represent an amino acid in the form of a feature vector. As a result, a sequence was transformed into a feature matrix filled with sequential feature vectors representing amino-acids.


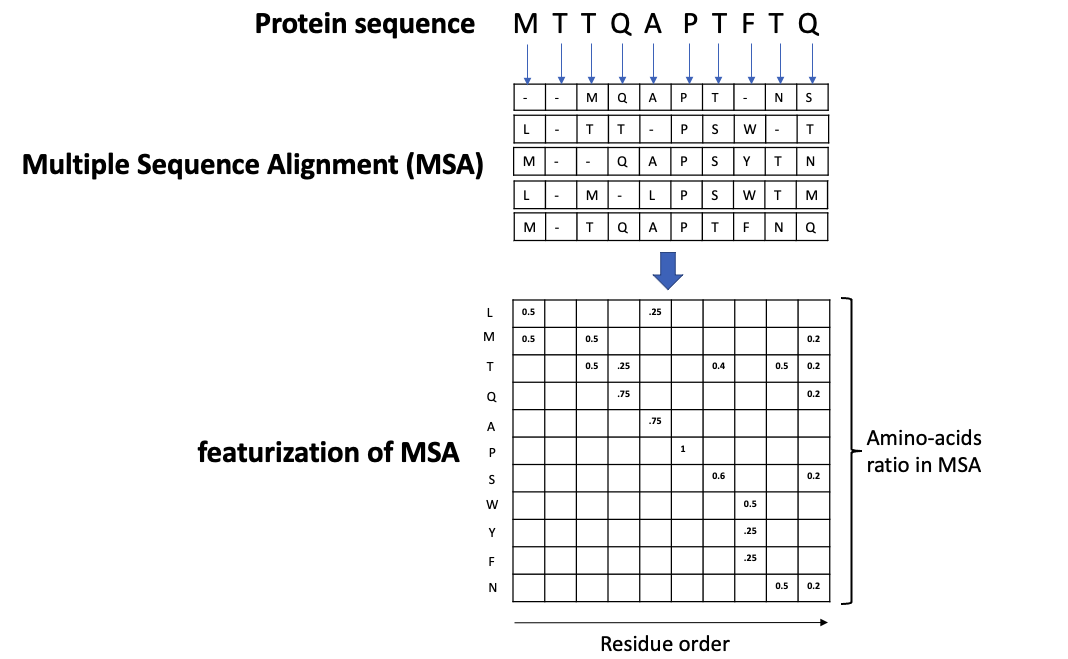


**Supplementary Figure 2. Transformation of MSA into a feature matrix.** We made use of conservation patterns in MSA as input features. As patterns in MSA can represent evolutionary constraints imposed on the residues of proteins, those features could be crucial for pathogenicity prediction. Each residue in the sequence was imposed with a vector containing the ratio of amino acids evolutionarily found in the residue based on MSA.

**Supplementary Table 2. Concordance between 3Cnet scores and revised ClinVar labels**

| Label Transition Type | Total | Benign (s < 0.25) | VUS (0.25 ≤ s < 0.75) | Pathogenic (s ≥ 0.75) |
| --- | --- | --- | --- | --- |
| Benign → Pathogenic | 0 | - | - | - |
| Benign → VUS | 0 | - | - | - |
| VUS → Pathogenic | 72 | 5 (6.9%) | 15 (20.8%) | **52 (72.2%)** |
| VUS → Benign | 169 | **141 (83.4%)** | 21 (12.4%) | 7 (4.1%) |
| Pathogenic → Benign | 1 | **1 (100%)** | 0 | 0 |
| Pathogenic → VUS | 0 | - | - | - |

3Cnet scores were able to correctly predict many of the ClinVar cases whose labels were revised between April 2020 and August 2020. 3Cnet scores less than 0.25 (s < 0.25) were binned as “benign,” those between 0.25 and 0.75 (0.25 ≤ s < 0.75) were binned as “variants of uncertain significance” (VUS), and those greater than or equal to 0.75 (s ≥ 0.75) were binned as “pathogenic.” Out of the consequence types examined (missense, start lost, stop gain, deletion, frameshift), only one case (NP_001035233.1:p.Arg607Gln) was found to have been revised from “pathogenic” to “benign”. 3Cnet successfully predicted the change. Cases revised from “benign” to “pathogenic”, “benign” to “VUS”, and “pathogenic” to “VUS” were not found.

**Supplementary Note 2. Difficulty in drawing PR-curves for SIFT and Polyphen2**

In the cases of SIFT and Polyphen2, precision-recall curves could not be drawn properly for patient data. It was because that too many variants for the data were scored as the highest pathogenicity (0 for SIFT, 1 for Polyphen2). 2,210 variants for SIFT and 3,451 variants for Polyphen2 were scored as the highest. However, among those variants only 58 and 61 variants were disease-causing variants and others were non-causal variants, respectively. It led to a sharp drop of precision rate from 1 to 0.026 for SIFT (from 1 to 0.018 for Polyphen2), which resulted in the straight line when drawing PR-curves (Supplementary Figure 3). Even though we could still calculate the area under curve, the metric might be highly distorted because the precision rates between those two points were completely missing.


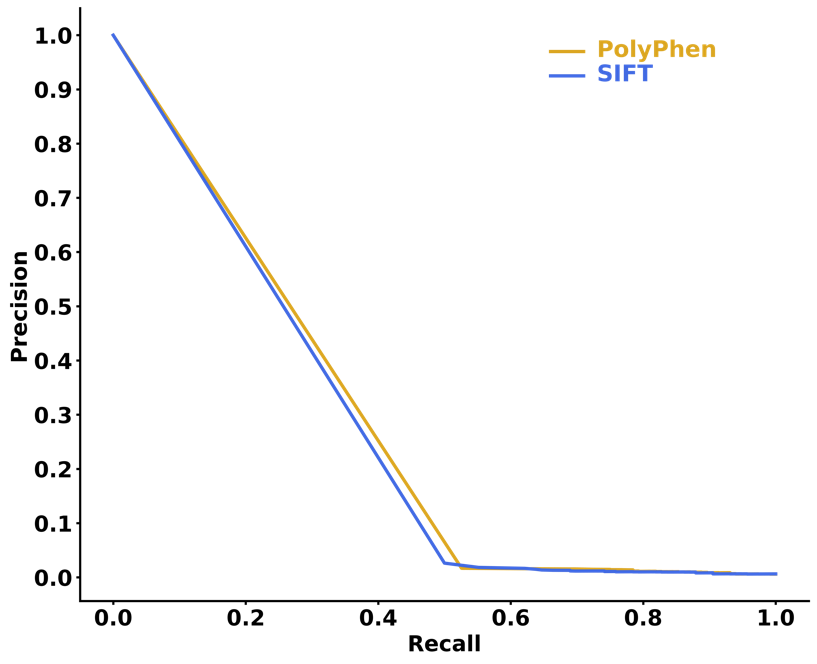


**Supplementary Figure 3. Precision-recall curves of SIFT and Polyphen2 for patient data.** 2,210 variants and 3,451 variants were scored as the highest pathogenicity for SIFT and Polyphen2, which led to sharp drops of precision rate and distorted curves.

**Supplementary Note 3. Determining the appropriate score threshold for 3Cnet scores** Using the scores for missense variants found in the genome of patients, we estimated the probability of the variants to be pathogenic for different score thresholds. From the cases where patients are diagnosed and the disease-causing variant(s) are known, rare missense variants (AF < 0.1 %) from the genome were collected. There were 186 disease-causing variants which were regarded as pathogenic variants. Other 3,720 missense variants were randomly selected as benign variants so that the prior probability could be 5%. We calculated the posterior probability based on the scores of those variants using the equation below:

$$P(pathogenic | score\geq T_{s})$$

where Ts indicates a score threshold. Also, we calculated the recall rate of pathogenic variants for the score threshold. When the score threshold of 3Cnet was set to 0.75, the probability of pathogenicity was 50.3 % and 70 % of pathogenic variants could be found. For the stringent threshold of 3Cnet scores, we recommend 0.87 because both the probability and the recall rate were above 60 % (60.5 % for probability and 60.2 % for recall rate). The probability and recall rate were plotted for different score thresholds for 3Cnet scores and REVEL scores, respectively (Supplementary figure 4). In the case of REVEL scores, the probability and the recall rate were 47.8 % and 59.7 % for the score threshold of 0.75.

**Supplementary Figure 4. The estimated probability of pathogenicity and the recall rate for different score thresholds.** 186 pathogenic variants and 3,720 benign missense variants from the genome of patients were used to estimate the probability of pathogenicity. For the incremental probability for the higher score, the probability between two thresholds were adjusted by linear interpolation when the probability was decreased. **a** The probability and recall rate for different score thresholds for 3Cnet scores. The probability increased rapidly for the score thresholds above 0.87, but the recall rate also dropped considerably. **b** The probability and recall rate for different score thresholds for REVEL scores.
