# Supplementary figures and images for: 3Cnet: pathogenicity prediction of human variants using multitask learning with evolutionary constraints
Source: Bioinformatics. 2021 Jul 16;37(24):4626–34. doi: 10.1093/bioinformatics/btab529 (PMC8665754; doi:10.1093/bioinformatics/btab529)

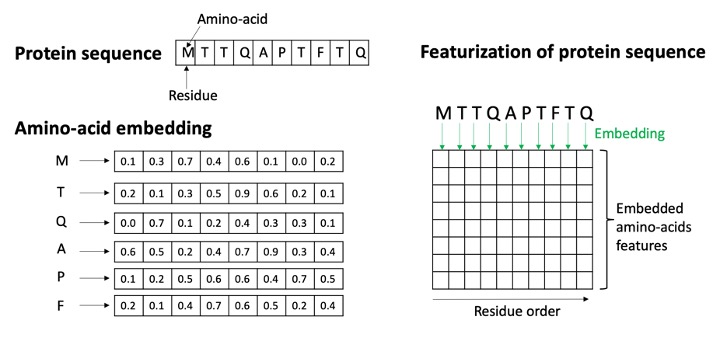

Supplement: btab529_Supplementary_Data [file btab529_supplementary_data.zip › sup_figure1.jpg]

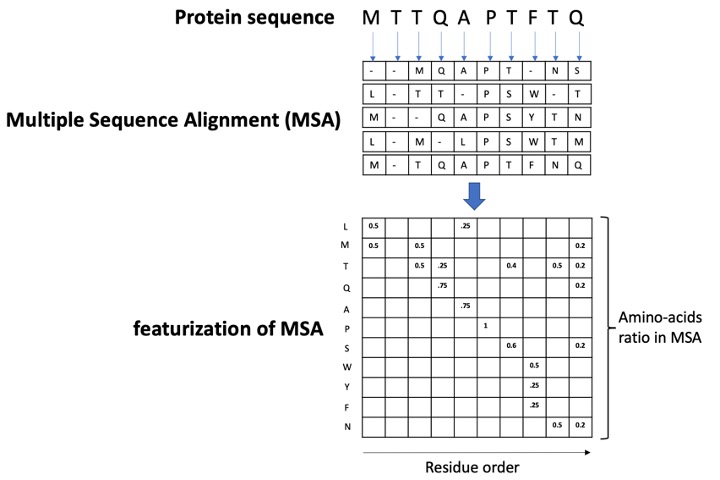

Supplement: btab529_Supplementary_Data [file btab529_supplementary_data.zip › sup_figure2.jpg]

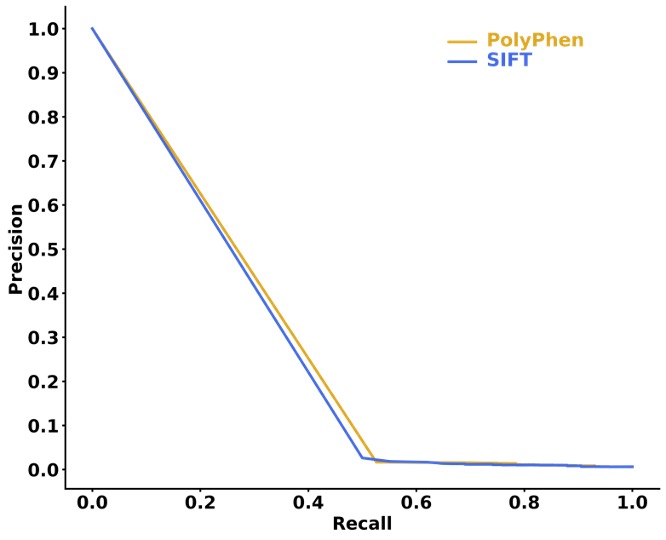

Supplement: btab529_Supplementary_Data [file btab529_supplementary_data.zip › sup_figure3.jpg]

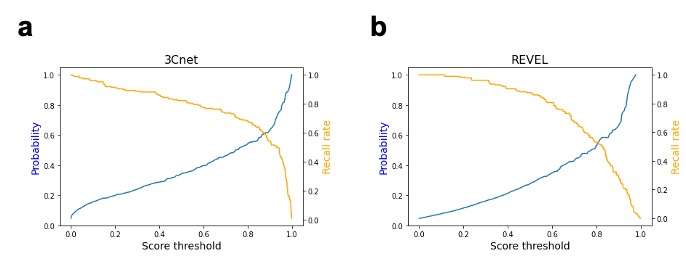

Supplement: btab529_Supplementary_Data [file btab529_supplementary_data.zip › sup_figure4.jpg]
